# Our Journal

**Published:** 1868-12

**Authors:** 


OUR JOURNAL.—We would most respectfully ask the coöperation of our subscribers and readers in extending our circulation, with the view that additions and improvements will be made as returns justify it. Reports of cases, items of medical news, and practical papers are earnestly solicited, and will be promptly acknowledged and early published. We hope to hear from our medical friends and have report of their most interesting and instructive experience. Our reliance is upon the profession; and from an experience of nearly eight years in journalism we now feel sure of support. The coming year appears to our journal full of promise, and we anticipate greater success, a wider circulation and higher standard of excellence. Will its friends aid in securing it?

